# Efficacy in Using Urodynamic Parameters of Intravesical Electrical Stimulation for Detrusor Underactivity

**DOI:** 10.5152/tud.2025.24181

**Published:** 2025-06-04

**Authors:** Anil Kmar, Aviral Sivastava, Rachana Tripathy, Lalit Kumar, Yashasvi Singh, Ujwal Kumar, Sameer Trivedi, S. N. Sankhwar

**Affiliations:** Department of Urology, Institute of Medical Sciences, Banaras Hindu University, Varanasi, India

To the Editor,

Subject: Clarification and Comments on the Article “Efficacy in Using Urodynamic Parameters of Intravesical Electrical Stimulation for Detrusor Underactivity”^1^

The article titled “Efficacy in Using Urodynamic Parameters of Intravesical Electrical Stimulation for Detrusor Underactivity” by Rahmat Aidil Fajar Siregar et al,^1^ published in *Urology Research and Practice* (2024) was recently reviewed. While the study presents valuable insights into the potential of intravesical electrical stimulation (IVES) for treating detrusor underactivity (DUA), there are a few points that merit further clarification and discussion:

1. Inclusion and Exclusion Criteria

The inclusion and exclusion criteria are pretty reasonable and exhaustive.

2. Gender-Specific Variability in DUA Definition

The definition of female DUA is not universally accepted because other authors use different criteria, and there are no known and universally accepted criteria yet. The diagnostic criteria for detrusor underactivity differ between male and female patients, as indicated by using distinct urodynamic parameters (bladder contractility index for males and Qmax for females).^2^ A more precise rationale for these differing criteria would strengthen the study’s conclusions.

3. Management of Bladder Outlet Obstruction (BOO)

It is not clear from the article how bladder outlet obstruction (BOO) was diagnosed in patients with concomitant DUA. There seems to be an error when the study authors stated that the first group had “prolonged BOO after treatment” instead of “prolonged BOO before treatment.”

4. Pharmacological Treatment for DUA

The study does not mention the pharmacological treatments prescribed to patients with DUA, if any. In clinical practice, muscarinic agonists and other pharmacotherapies may be used to manage symptoms of DUA, though not entirely practical.^3^

5. Improvement in IPSS Score

While the study discusses and mentions changes in urodynamic parameters following IVES very well, it would have been better to see improvement in the International Prostate Symptom Score (IPSS) or other relevant symptom assessment scales with IVES.

6. Low Number of CIC-Independent Patients Post-IVES

The study mentions that a few patients became independent from clean intermittent catheterization (CIC) following IVES therapy, with only 7 out of 56 patients becoming independent. A difference of 1-2 mmHg does not seem clinically significant. The low number of CIC-independent patients raises questions about the efficacy of IVES in clinical relevance.

Finally, I would like to highlight another significant limitation of the study: the lack of a control group, which leads to biases in the study, such as the placebo effect, regression to the mean, Hawthorne effect, or natural evolution.

In conclusion, while this study adds essential knowledge and insight to the management of DUA with IVES, I believe addressing the above points would provide greater clarity and enhance the overall interpretation of the findings. I look forward to further research in this area and hope that future studies will be carried out to validate the present research results and provide additional insights into the optimal management strategies for patients with DUA.

## Data Availability

The data that support the findings of this study are available on request from the corresponding author.

